# Admission NarxCare Narcotic Scores Are Associated With Increased Odds of Readmission and Prolonged Length of Hospital Stay After Primary Elective Total Knee Arthroplasty

**DOI:** 10.5435/JAAOSGlobal-D-22-00040

**Published:** 2022-12-05

**Authors:** Anoop R. Galivanche, Justin Zhu, Michael R. Mercier, Ryan McLean, Christopher V. Wilhelm, Arya G. Varthi, Jonathan N. Grauer, Lee E. Rubin

**Affiliations:** From the Department of Orthopaedics and Rehabilitation, Yale School of Medicine, New Haven, CT.

## Abstract

The association of preoperative narcotic use with postoperative outcomes after primary elective total knee arthroplasty (TKA) has remained poorly characterized. The NarxCare platform analyzes patients' state Prescription Drug Monitoring Program records to assign numerical scores that approximate a patient's overall opioid usage. The present study investigated the utility of admission NarxCare narcotic scores in predicting the odds of adverse events (AEs) after primary elective TKA.

**Methods:**

Elective primary TKA patients performed at a single institution between October 2017 and May 2020 were evaluated. NarxCare narcotic scores at the time of admission, patient characteristics, 30-day AEs, readmissions, revision surgeries, and mortality were abstracted. Elective TKA patients were binned based on admission NarxCare narcotic scores. The odds of experiencing adverse outcomes were compared.

**Results:**

In total, 1136 patients met the criteria for inclusion in the study (Narx Score 0: n = 293 [25.8%], 1 to 99: n = 253 [22.3%], 100 to 299: n = 368 [32.4%], 300 to 499: n = 161 [14.2%], and 500+: n = 61 [5.37%]). By logistic regression, patients with higher admission narcotic scores tended to have a dose-dependent increase in the odds of prolonged length of hospital stay, readmission within 30 days, and aggregated AEs.

**Discussion:**

Admission narcotic scores may be used to predict readmission and to stratify TKA patients by risk of AEs.

In the United States, rates of prescription opioid utilization have increased substantially over the past several decades.^1^ Although alternatives are becoming increasingly widespread, opioids remain a common form of management for knee pain.^2-4^ As the opioid epidemic has unfolded, the use of such medications has become a focal point of medical and legislative reform in an effort to curb the rising prevalence of opioid-related mortality.^1,5^

Several studies have reported preoperative opioids to be associated with adverse perioperative outcomes after total knee arthroplasty (TKA).^6,7,8,9^ Rozell et al.^7^ and Chen et al.^6^ reported increases in length of stay, whereas Bedard et al^8^ reported greater risk for early revision. The study by Rozell et al. used data in the electronic medical record (EMR) to measure opioid consumption.^10^ Typically collected by patient self-reported opioid use, such data are potentially subject to response bias or erroneous reporting. Chen et al. and Bedard et al. used administrative claims data to measure opioid consumption, which can be limited by recording biases, clerical errors, and temporal changes in coding.^11,12^ These studies did not examine the relationship between preoperative narcotic use and differential patient satisfaction after joint surgery. Prior evidence has remained limited to spine surgery literature.^13^

Many institutions now use state pharmacy databases to monitor narcotic use by their patients. One such database is the Prescription Drug Monitoring Program, which requires all pharmacies to routinely report prescription data for scheduled medications, including opioids.^13^ NarxCare (Appriss Health) is a platform that facilitates the use of state databases and then quantifies a score for overall opioid and benzodiazepine medication use through a proprietary tool.

The NarxCare narcotic score is weighted for recent activity and is influenced by the following parameters: number of prescribers, number of pharmacies, morphine milligram equivalents, and overlapping prescriptions for each patient.^13^ Scores range from 0 (suggesting opioid naivete) to 999, with higher numbers for each aforementioned parameter, resulting in a higher NarxCare narcotic score.

The present study explores the predictive utility of the Narx Score system for complications after primary TKA. To accomplish this investigation, we examined a cohort of 1,136 elective TKA procedures performed at our institution between October 2017 and May 2020.

## Methods

### Study Population

An orthopaedic surgery registry was retrospectively compiled at a large academic medical center that included data on patient characteristics, 30-day adverse events (AEs), readmissions, revision surgeries, and mortality. This institutional registry additionally included Narx Score data, which is a component of the NarxCare platform. The NarxCare platform for Prescription Drug Monitoring Program analytics is integrated directly into the EMR system (Epic Systems) at our institution. Our institution's Human Investigations Committee exempted this study from further review based on its retrospective design that did not involve any further patient contact after the collection and deidentification of data.

Elective TKA patients from October 2017 to May 2020 were identified within the registry using the Current Procedural Terminology code 27447. Nonelective patients were excluded from the study. Patients with incomplete or missing demographic, comorbidity, or 30-day perioperative outcome data were further excluded from the study.

### Cohort Characteristics

The following preoperative variables were extracted from the EMR: age, sex, weight and height (used to calculate the body mass index [BMI]), preoperative functional status, and American Society of Anesthesiology (ASA) classification.

The NarxCare narcotic score on admission was then extracted for each of the patients. Narx Scores range from 0 to 999, with the last digit of the score being the number of active narcotic scripts prescribed to a patient at a given time. These scores were binned into the following categories: 0, 1 to 99, 100 to 299, 300 to 499, and greater than 500.

### Postoperative Outcomes

Postoperative variables included total length of hospital stay (LOS), 30-day AEs, readmissions, and revision surgeries. These were collected per the National Surgery Quality Improvement Program guidelines.

Perioperative AEs were classified as serious adverse events (SAEs) or minor adverse events (MAEs). An SAE was defined as the occurrence of any of the following: deep surgical site infection, sepsis, failure to wean, unplanned reintubation, postoperative renal failure, deep vein thrombosis, pulmonary embolism, cardiac arrest, myocardial infarction, or stroke. An MAE was defined as the occurrence of any of the following: superficial surgical site infection, wound dehiscence, pneumonia, urinary tract infection, or postoperative renal insufficiency. Patients that experienced either an SAE or MAE were further classified as having experienced any adverse event (AAE). Readmission and revision surgery within 30 days were collected as additional variables.

### Statistical Analysis

The demographic characteristics, LOS, occurrence of AEs, readmission rate, revision surgery rate, and mortality of the different narcotic score cohorts were first compared. Demographic characteristics between the different cohorts were compared using chi-square tests. Total LOS was compared between the different cohorts using analysis of variance (ANOVA).

Multivariable logistic regression was used to compare the occurrence of AEs in the different narcotic score cohorts. Multivariable regressions controlled for age, sex, BMI, preoperative functional status, and ASA classification.

The 0 narcotic score cohort was used as the control group to identify opioid-naive TKA patients in these regressions. All statistical analyses were performed using STATA version 17 (StataCorp LP).

## Results

### Cohort Characteristics

**Figure 1:**
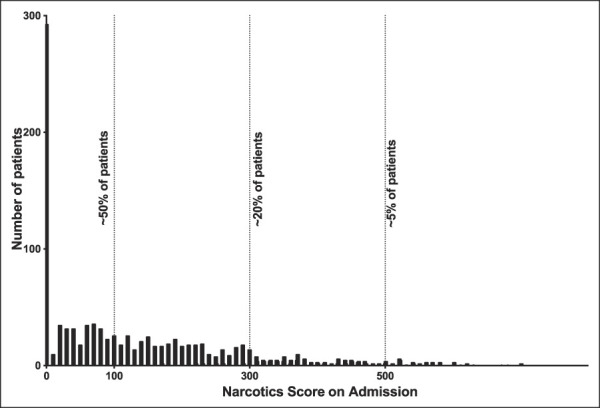
Histogram showing the distribution of NarxCare narcotic scores on admission for elective total knee arthroplasty (TKA) in the study sample.

In total, 1,136 patients met the criteria for inclusion in the study population. Of the total study population, 293 patients (25.79%) had an admission narcotic score of 0, 253 patients (22.27%) had an admission narcotic score between 001 and 099, 368 patients (32.39%) had an admissions narcotic score between 100 and 299, 161 patients (14.17%) had an admission narcotic score between 300 and 499, and 61 patients (5.37%) had an admission narcotic score of 500 or greater (Table 1 and Figure 1).

**Table 1:** Demographic and Comorbid Characteristics of the Study Population

Narcotic Score on Admission	Total Study Population		0		1-99		100-299		300-499		500+		
	Number	Percent	Number	Percent	Number	Percent	Number	Percent	Number	Percent	Number	Percent	*P* value^a^
Total no. of patients	1136	100.00	293	25.79	253	22.27	368	32.39	161	14.17	61	5.37	
Age (yr, mean ± SD)	65.2 ± 9.9		67.3± 10.0		65.8 ± 8.8		64.6 ± 9.6		64.0 ± 10.4		59.1 ± 11.2		**<0.001**
≤40	14	1.23	0	0.00	1	0.40	7	1.90	3	1.86	3	4.92	
41-50	51	4.49	11	3.75	9	3.56	16	4.35	6	3.73	9	14.75	
51-60	264	23.24	60	20.48	50	19.76	84	22.83	51	31.68	19	31.15	
61-70	461	40.58	106	36.18	115	45.45	161	43.75	58	36.02	21	34.43	
71-80	278	24.47	86	29.35	65	25.69	86	23.37	33	20.50	8	13.11	
>80	68	5.99	30	10.24	13	5.14	14	3.80	10	6.21	1	1.64	
BMI (kg/m^2^, mean ± SD)	33.4 ± 7.3		32.3 ± 6.9		33.3 ± 6.4		34.2 ± 7.7		34.4 ± 7.4		31.2 ± 7.8		**<0.001**
<18.5	6	0.53	2	0.68	0	0.00	1	0.27	2	1.24	1	1.64	** **
18.5-25	102	8.98	34	11.60	16	6.32	33	8.97	7	4.35	12	19.67	
25-30	302	26.58	93	31.74	65	25.69	88	23.91	40	24.84	16	26.23	
30-40	531	46.74	121	41.30	137	54.15	174	47.28	76	47.20	23	37.70	
40-50	168	14.79	38	12.97	30	11.86	59	16.03	34	21.12	7	11.48	
≥50	27	2.38	5	1.71	5	1.98	13	3.53	2	1.24	2	3.28	
Sex													0.066
Female	724	63.73	179	61.09	154	60.87	257	69.84	97	60.25	37	60.66	
Male	412	36.27	114	38.91	99	39.13	111	30.16	64	39.75	24	39.34	
Functional status before surgery													0.225
Independent	1105	97.27	289	98.63	248	98.02	356	96.74	154	95.65	58	95.08	
Part/total dependent	31	2.73	4	1.37	5	1.98	12	3.26	7	4.35	3	4.92	
ASA													**0.001**
1	17	1.50	8	2.73	4	1.58	3	0.82	1	0.62	1	1.64	
2	478	42.08	144	49.15	122	48.22	137	37.23	51	31.68	24	39.34	
3+	641	56.43	141	48.12	127	50.20	228	61.96	109	67.70	36	59.02	
	Mean	SD	Mean	SD	Mean	SD	Mean	SD	Mean	SD	Mean	SD	*P* value^a^
Length of stay (d)	2.6	2.1	2.4	1.1	2.6	1.4	2.6	1.1	3.0	4.7	3.1	1.5	**0.030**

a *P* < 0.05. Bolded p-values are statistically significant at *p*<0.05.

The average age of patients in the study population was 65.2 ± 9.9 (mean ± SD) years. There was a statistically significant difference in age between patients in the different admission Narx Score groups (higher Narx Score groups associated with lower age, *P* < 0.001). The average BMI of patients in the study cohort was 33.4 ± 7.3 kg/m^2^, and there were statistically significant differences in BMI between patients in the different admission Narx Score groups (*P* < 0.001) by ANOVA. There were no statistically significant differences in sex or functional status between the admission Narx Score groups (Table 1).

In terms of ASA, there were differences between Narx Score groups by ANOVA (*P* = 0.001). The opioid-naive group had the lowest percentage of ASA 3+ patients (48.12%), and the 300 to 499 group had the highest percentage of ASA 3+ patients (67.70%).

### Postoperative Outcomes

The mean total LOS of the study population was 2.6 ± 2.1 days. The mean length of stay increased with increasing admission Narx Score group (*P* = 0.030, Table 1).

The rate of AAEs in the total study population within 30 days of the index procedure was 5.63%, the rate of SAEs in the total study population was 3.35%, and the rate of MAEs in the total study population was 2.99%. These findings are summarized by the Narx Score group in Table 2. On univariate analysis, rates of AAE, SAE, and MAE increased with increasing admission Narx Score group (*P* = 0.001, *P* < 0.001, and *P* = 0.001, respectively).

**Table 2:** Number of Adverse Events, Return to the Operating Room, Readmissions, and Mortality for Patients of Varying Narcotic Scores

Narcotic Score on Admission	Total study population		0		1-99		100-299		300-499		500+		
Total Number of Patients	n = 1136		n = 293		n = 253		n = 368		n = 161		n = 61		^a^*P* value
	Number	Percent	Number	Percent	Number	Percent	Number	Percent	Number	Percent	Number	Percent	
Any adverse event (AAE)	64	5.63	17	5.80	6	2.37	23	6.25	26	16.15	13	21.31	**0.001**
Serious adverse event (SAE)	38	3.35	8	2.73	4	1.58	12	3.26	10	6.21	4	6.56	**<0.001**
Deep infection	4	0.35	0	0.00	1	0.40	1	0.27	2	1.24	0	0.00	
Sepsis	7	0.62	2	0.68	0	0.00	0	0.00	4	2.48	1	1.64	
Failure to wean	1	0.09	0	0.00	0	0.00	0	0.00	1	0.62	0	0.00	
Reintubation	2	0.18	0	0.00	0	0.00	0	0.00	1	0.62	1	1.64	
Renal failure	1	0.09	0	0.00	0	0.00	0	0.00	1	0.62	0	0.00	
Thromboembolic events	23	2.02	5	1.71	3	1.19	9	2.45	4	2.48	2	3.28	
Cardiac arrest	0	0.00	0	0.00	0	0.00	0	0.00	0	0.00	0	0.00	
MI	4	0.35	1	0.34	0	0.00	0	0.00	3	1.86	0	0.00	
Stroke	3	0.26	1	0.34	0	0.00	2	0.54	0	0.00	0	0.00	
Minor adverse event (MAE)	34	2.99	10	3.41	3	1.19	6	1.63	9	5.59	6	9.84	**0.001**
Superficial infection	12	1.06	3	1.02	2	0.79	4	1.09	0	0.00	3	4.92	
Dehiscence	0	0.00	0	0.00	0	0.00	0	0.00	0	0.00	0	0.00	
Pneumonia	8	0.70	1	0.34	1	0.40	1	0.27	4	2.48	1	1.64	
UTI	13	1.14	6	2.05	0	0.00	1	0.27	5	3.11	1	1.64	
Renal insufficiency	4	0.35	1	0.34	0	0.00	0	0.00	2	1.24	1	1.64	
Return to the operating room within 30 d of operation	15	1.32	5	1.71	3	1.19	3	0.82	4	2.48	0	0.00	0.466
Readmission within 30 d of operation	70	6.16	12	4.10	10	3.95	24	6.52	16	9.94	8	13.11	**0.009**
Mortality within 30 d of operation	0	0.00	0	0.00	0	0.00	0	0.00	0	0.00	0	0.00	1.000

a *P* < 0.05. MI = myocardial infarction, UTI = urinary tract infection.

**Figure 2:**
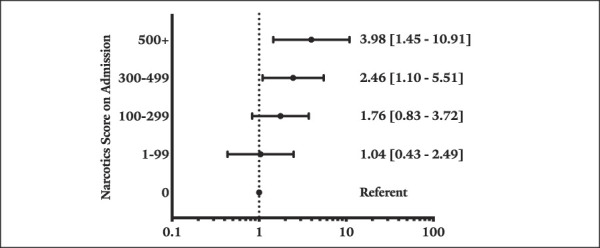
Forest plot illustrating odds ratios for readmission within 30 days of the index procedure, using the NarxCare narcotic score 0 group as the referent.

On multivariable logistic regression assessing AEs, the highest Narx Score group had increased odds of AAE compared with the opioid-naive group (odds ratio [OR] = 2.96; 95% confidence interval [CI] = 1.18 to 7.40; *P* = 0.021). There were no other statistically significant differences in aggregated AEs between the different Narx Score groups (*P* > 0.05 for all). Table 3 and Figure 2 show the findings of the multivariable logistic regression analyses. Multivariable regressions that treated the admission Narx Score as a continuous variable did not find admission Narx Scores to be associated with postoperative complications.

**Table 3:** Multivariate Odds Ratio for Adverse Events, Return to the Operating Room, Readmissions, and Mortality for Patients of Varying Narcotic Scores

Narcotic Score on Admission	0		1-99		100-299		300-499		500+	
Total Number of Patients (n = 1136)	n = 293		n = 253		n = 368		n = 161		n = 61	
	Odds Ratio	^a^*P* value	Odds Ratio	^a^*P* value	Odds Ratio	^a^*P* value	Odds Ratio	^a^*P* value	Odds Ratio	^a^*P* value
Any adverse event (AAE)	1	1.000	0.39	0.051	0.80	0.543	1.39	0.399	**2.96**	**0.021**
Serious adverse event (SAE)	1	1.000	0.57	0.371	1.21	0.689	2.24	0.103	2.75	0.122
Minor adverse event (MAE)	1	1.000	0.31	0.084	0.41	0.103	1.41	0.487	3.06	0.056
Revision surgery within 30 d of operation	1	1.000	0.72	0.656	0.50	0.355	1.39	0.634	N/A	N/A
Readmission within 30 d of operation	1	1.000	1.04	0.931	1.76	0.139	**2.46**	**0.029**	**3.98**	**0.007**

a *P* < 0.05.

Controlled for age, sex, BMI, functional status, and ASA classification.

Return to the operating room within 30 days of surgery was noted for 1.32% of the study population, and none of the patients died. There were no differences in the odds of revision surgery on multivariable regression by admission Narx Score group. There were insufficient events for the analysis of mortality on multivariable regression.

Readmission to the hospital within 30 days of surgery was noted for 6.16% of the study population. The rates of readmission increased with increasing admission Narx Score group (*P* = 0.009). On multivariable regression, patients in the following admission Narx Score groups had higher odds of readmission: 300 to 499 (OR = 2.46; 95% CI, 1.10 to 5.51; *P* = 0.029) and 500+ (OR = 3.98; 95% CI, 1.45 to 10.91; *P* = 0.007).

## Discussion

Previous studies have noted that preoperative opioid use is associated with adverse perioperative outcomes after TKA, including in-hospital complications and greater risk for early revision.^6,7^ The current study probes whether preoperative opioid use (as measured by the NarxCare narcotic score) is associated with postoperative outcomes after TKA.

The current study found that younger patients tended to have higher preoperative NarxCare narcotic scores. This is consistent with the findings of Goesling et al.^14^ The current study also found that preoperative Narx Scores were higher for those with higher ASA classification. This is consistent with a previous study by Bedard et al, which found that several comorbidities (ie, anxiety/depression, low back pain, myalgia, and drug/alcohol/smoking dependence) were associated with an increased risk of opioid use.^3^ Taken together, these findings indicate that the groups with higher Narx Scores are younger and less healthy.

Univariable analyses found that the higher Narx Score groups had increased rates of any, serious, and minor AEs. The univariate ANOVA findings are consistent with those of Kim et al., which found that patients with consistent preoperative opioid use were at higher risk of several types of perioperative AEs compared with opioid-naive patients.^9^ However, once Kim et al adjusted their results for differences in demographics and preoperative comorbidity burden, they no longer observed differential risk of 30-day mortality. These results indicate that the association between preoperative opioid use and AEs may be confounded by a higher rate of preoperative comorbidities in narcotic users. These results suggest that further investigation of preoperative patient characteristics may be warranted to improve perioperative outcomes.

Both univariate and multivariable analyses found that the two highest narcotic score groups had a significantly higher risk of readmission (ORs of 2.46 and 3.98) within 30 days of TKA compared with the other groups. These findings are consistent with Weick et al., who found that preoperative opioid use increased 30-day readmission rates after TKA.^15^ The results of our study have implications for how narcotic scores can be used to assist and inform healthcare policy, as readmission rate is a critical factor in hospital reimbursement. Readmission rate became linked to hospital reimbursement in 2012 when the Affordable Care Act was passed with the Hospital Readmissions Reduction Program (HRRP).^16^ The HRRP financially penalizes hospitals for excessive readmissions for certain medical conditions and surgeries, specifically including TKA and total hip arthroplasty [B].^17^ The HRRP was aimed to incentivize hospitals to reduce costs and improve patient care, as 9% to 48% of readmissions are thought to be preventable.^18–20^ In response to these changes, hospitals have strived to reduce the readmission rate through several means.^17^ As they embark on these initiatives, hospitals can readily benefit from tools that can meaningfully identify patients' risk levels for readmission and personalize care. Our study indicates that the Narx Score has the potential to become one such tool and thus merits further exploration. Continued evaluation of this new scoring tool is warranted as it becomes increasingly adopted nationally.

The current study has several limitations. First, the NarxCare narcotic scores used to approximate opioid burden have not been previously validated and may not be available at all hospitals. In addition, these scores are proprietary, and the exact scoring algorithm is not publicly known. Second, Physician Drug Monitoring Program data can only track how often a prescription was filled but cannot track how much medication a patient took. Third, the present study was performed at a single institution, potentially limiting its generalizability.

The present study sheds light on the relationship between Narx Scores and both perioperative and postoperative outcomes. Patients with higher Narx Scores had increased odds of readmission, prolonged LOS, and aggregated AEs. In addition, this study identified key differences in demographics and comorbidities between Narx Score groups. These demographic and comorbid variations may account for the disparity in perioperative outcomes as initially identified by the univariate analysis. As a result, this may warrant further examination of preoperative patient characteristics to optimize patient outcomes through the identification of at-risk populations^1^ and personalizing treatment for these patients.
